# Delineation of recurrent glioblastoma by whole brain spectroscopic magnetic resonance imaging

**DOI:** 10.1186/s13014-023-02219-2

**Published:** 2023-02-22

**Authors:** Jonathan B. Bell, William Jin, Mohammed Z. Goryawala, Gregory A. Azzam, Matthew C. Abramowitz, Tejan Diwanji, Michael E. Ivan, Maria del Pilar Guillermo Prieto Eibl, Macarena I. de la Fuente, Eric A. Mellon

**Affiliations:** 1 Department of Radiation Oncology, Sylvester Comprehensive Cancer Center, Miller School of Medicine, University of Miami, 1475 NW 12th Ave, Miami, FL 33136 USA; 2 Department of Radiology, Miller School of Medicine, University of Miami, Miami, FL USA; 3 Department of Neurological Surgery, Miller School of Medicine, University of Miami, Miami, FL USA; 4 Department of Neurology and Sylvester Comprehensive Cancer Center, Miller School of Medicine, University of Miami, Miami, FL USA

**Keywords:** Recurrent glioblastoma, Spectroscopic MRI, Re-irradiation, Glioblastoma

## Abstract

**Background:**

Glioblastoma (GBM) cellularity correlates with whole brain spectroscopic MRI (sMRI) generated relative choline to *N*-Acetyl-Aspartate ratio (rChoNAA) mapping. In recurrent GBM (rGBM), tumor volume (TV) delineation is challenging and rChoNAA maps may assist with re-RT targeting.

**Methods:**

Fourteen rGBM patients underwent sMRI in a prospective study. Whole brain sMRI was performed to generate rChoNAA maps. TVs were delineated by the union of rChoNAA ratio over 2 (rChoNAA > 2) on sMRI and T1PC. rChoNAA > 2 volumes were compared with multiparametric MRI sequences including T1PC, T2/FLAIR, diffusion-restriction on apparent diffusion coefficient (ADC) maps, and perfusion relative cerebral blood volume (rCBV).

**Results:**

rChoNAA > 2 (mean 27.6 cc, range 6.6–79.1 cc) was different from other imaging modalities (*P* ≤ 0.05). Mean T1PC volumes were 10.7 cc (range 1.2–31.4 cc). The mean non-overlapping volume of rChoNAA > 2 and T1PC was 29.2 cm^3^. rChoNAA > 2 was 287% larger (range 23% smaller–873% larger) than T1PC. T2/FLAIR volumes (mean 111.7 cc, range 19.0–232.7 cc) were much larger than other modalities. rCBV volumes (mean 6.2 cc, range 0.2–19.1 cc) and ADC volumes were tiny (mean 0.8 cc, range 0–3.7 cc). Eight in-field failures were observed. Three patients failed outside T1PC but within rChoNAA > 2. No grade 3 toxicities attributable to re-RT were observed. Median progression-free and overall survival for re-RT patients were 6.5 and 7.1 months, respectively.

**Conclusions:**

Treatment of rGBM may be optimized by sMRI, and failure patterns suggest benefit for dose-escalation within sMRI-delineated volumes. Dose-escalation and radiologic-pathologic studies are underway to confirm the utility of sMRI in rGBM.

## Background

Standard of care for primary glioblastoma (GBM) consists of maximally safe surgical resection followed by concurrent chemoradiation and adjuvant chemotherapy [1, 2]. Despite these therapies, GBM carries a poor prognosis with a median survival of approximately 16–21 months [3]. There is significant interest in escalating radiotherapy (RT) dose in GBM to improve control, but targeting is limited by conventional MRI [4]. For example, T1 post-contrast (T1PC) enhancement only identifies the highest density tumor with neovascularization. Meanwhile, T2 Fluid Attenuated Inversion Recovery (T2/FLAIR) hyperintense volumes are non-specific and can correspond to gross tumor without neovascularization, areas of cellular infiltration disrupting normal brain architecture, benign vasogenic edema, leukoencephalopathy, or other changes [5]. Microscopic GBM infiltration can also extend outside of the FLAIR changes [6, 7].

Alternatively, spectroscopic MRI (sMRI) is an emerging technique performed with standard 3 T MRI hardware that maps certain endogenous compounds within a high percentage of the human brain without injected contrast [8]. Visualized metabolites include choline (Cho), a neuronal cell membrane component often elevated in actively dividing tumors [9, 10]. *N*-Acetyl-Aspartate (NAA) is an intermediary in many neuronal metabolic processes but is decreased with neuronal dysfunction or replacement [11]. Cho:NAA ratio was found to best correlate to GBM cellularity on targeted biopsies compared to other MRI metrics [7]. A relative Cho:NAA ratio > 2 (rChoNAA > 2) normalized to contralateral white matter was found to best predict for presence of tumor, correlate with worse survival, and earlier recurrence [12–14].

These findings led to a multi-institutional pilot study using sMRI integrated into an RT planning workflow to guide radiotherapy dose escalation [15]. Thirty patients with newly-diagnosed GBM were treated using a simultaneous integrated boost (SIB) technique to a total dose of 75 Gy to the union of residual T1 post contrast (TIPC) enhancement and the rChoNAA ≥ 2 along with concurrent temozolomide (TMZ) [16]. Compared with historical data, the study showed favorable outcomes with a median OS of 23.0 months and a median PFS of 16.6 months. The toxicity profile was also acceptable, with most toxicity attributed to TMZ. The French SPECTRO-GLIO trial is also actively exploring the use of rChoNAA > 2 to delineate target volumes for dose escalation in the first-line setting [17].

Accurate target delineation in recurrent GBM (rGBM) is more challenging because of confounding effects from the primary treatment. For example, radiation-induced leukoencephalopathy or benign vasogenic edema can greatly exaggerate T2/FLAIR hyperintense volumes while radiation necrosis can mimic active disease on T1PC [18]. This volume uncertainty and fear of large volume re-irradiation (re-RT) toxicity in rGBM leads to gross tumor volume (GTV) based only on T1PC imaging with limited or no clinical target volume (CTV) [19–21]. Preliminary results from RTOG 1205 have identified a borderline progression free survival benefit to re-irradiation when added to bevacizumab, which suggests that improvements in re-irradiation could enhance survival [21].

Given the challenges of target delineation of rGBM for re-RT and the known utility of sMRI for first-line rGBM RT targeting, we hypothesized that rChoNAA would reveal clinically meaningful volumes of rGBM not detected with standard MRI. Improvements in rGBM definition by sMRI could lead to more accurate re-RT fields and lead to dose escalation trials for the highest risk areas.

## Methods

### Patient selection and characteristics

We analyzed 14 sequential rGBM patients who underwent sMRI. Patients were included if they had a pathologic diagnosis of glioblastoma (based on 2016 WHO criteria) or cIMPACT-NOW update 3 criteria for molecular features of glioblastoma, received one course of conventionally fractionated RT as part of combined modality treatment as first line treatment, and had multi-disciplinary consensus agreement for disease progression [22, 23]. Such consensus was based on progression of enhancing disease meeting at least one criteria: outside a prior treatment field, positive relative cerebral blood volume (rCBV) on perfusion MRI, increased ^18^FDG uptake on ^18^FDG-PET, or repeat resection or biopsy demonstrating GBM.

### Image acquisition and target delineation

MRI and MRSI data were acquired at 3 T (Siemens Medical Solutions, Erlangen, Germany). T1-weighted imaging was carried out using a 3D Magnetization Prepared Rapid Acquisition Gradient Echo sequence with 0.9 × 0.9 × 0.7 mm^3^ resolution; TR/TE/TI = 2300/2.41/930 ms; flip angle 9°; and image matrix 320 × 216 × 192. The protocol also included fluid-attenuated inversion recovery (FLAIR) (TR/TE = 9000/106 ms, resolution = 0.36 × 0.36 × 3 mm^3^, flip angle = 120°), and T2-weighted (TR/TE = 4810/76 ms, resolution = 0.45 × 0.45 × 3 mm^3^, flip angle = 160°) images and DTI acquired with TR/TE of 6300/99 ms and a resolution 1.5 × 1.5 × 3 mm^3^. Diffusion-sensitizing gradient encoding with diffusion weighting factor of b = 1000 s/mm^2^ was applied in 30 directions along with 9 averages for b = 0 s/mm^2^ (B0).

Whole brain sMRI was acquired using a spatial-spectral echo-planar readout with spin-echo excitation; TR/TE = 1551/50.0 ms; non-selective lipid inversion-nulling with TI = 198 ms; a field-of-view 280 × 280 × 180 mm^3^; matrix size 50 × 50 × 18 slices with elliptical k-space encoding; echo train length of 1000 points; bandwidth of 2500 Hz. The acquisition time was 15 min. Data was acquired using spatial oversampling with a nominal voxel volume of 0.313 cc [24, 25]. Post-processing resulted in a working voxel size of 4.4 mm × 4.4 mm × 5.6 mm.

**Fig. 1:**
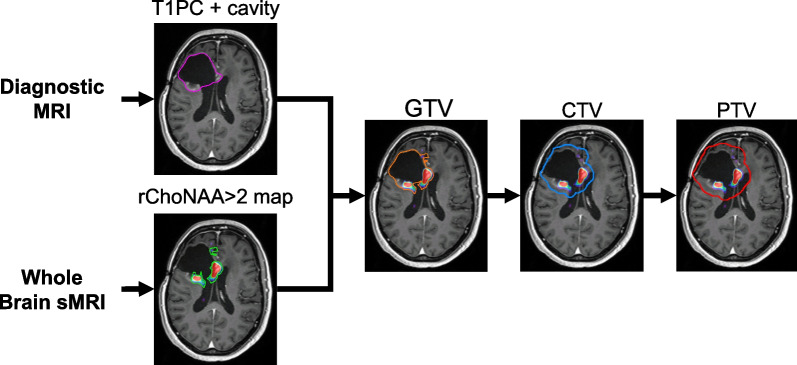
Whole brain sMRI workflow and contour generation. Following surgical resection, a diagnostic MRI was obtained, and the residual post-contrast enhancing volume and cavity were contoured (T1PC + cavity). Whole brain sMRI was performed and co-registered to the diagnostic MRI. rChoNAA > 2 maps were generated using the MIDAS software and contoured in the treatment planning software. The GTV was defined as the union of the T1PC + cavity and the rChoNAA > 2 map. CTV (3 mm) and PTV (3 mm) expansions were then generated

sMRI and non-contrast T1 sequences were converted into co-registered spatial-spectral data using the MIDAS software suite (University of Miami, Miami, FL) [25]. Residual contrast-enhancing volumes (T1PC volumes) were derived from the post-contrast T1-weighted imaging, which was outlined semi-automatically. Volumes corresponding to rChoNAA > 2 were identified as regions within the gross tumor region that had a Cho/NAA ratio greater than 2.0 relative to the mean of the Cho/NAA value in contralateral white matter [7, 26]. These rChoNAA > 2 and T1PC volumes were transferred to MIM for radiation contouring (MIM Software Inc; Beachwood, OH). A composite GTV was created as a union of rChoNAA > 2 on sMRI, T1PC enhancement, and resection cavity (if present) and expanded 3 mm to CTV (Fig. 1).

### Recurrent glioblastoma management

After imaging completed, patients and physicians were given the discretion as to whether to use the acquired data for therapy. Eleven of 14 patients decided to proceed with re-RT, and these cases were treated with the CTV as described above. Radiation doses of 35 Gy in 10 fractions were delivered either by intensity-modulated proton therapy (CTV with robustness 3 mm geometric uncertainty and 3.5% range uncertainty) or intensity-modulated photon RT (prescribed to CTV with additional 3 mm planning target volume). Nine of 11 patients received concurrent bevacizumab at 10 mg/kg every 2 weeks until progression or intolerance. Patients were seen for follow-up every 2 months thereafter with multiparametric MRI including T1PC, T2/FLAIR, rCBV, and apparent diffusion coefficient. Maximum allowed doses to 0.03 cc of the brainstem and optic structures were < 30 Gy and < 24 Gy, respectively.

### Patterns of failure analysis

Recurrence after re-RT was defined as described in the patient selection criteria, or, in the case of indeterminate imaging findings, a combination of imaging and evidence of clinical progression. If still unclear, cases were individually reviewed in a multidisciplinary oncology setting. In-field recurrences were defined as disease within the CTV. Marginal recurrences were defined as disease within 2 cm of the CTV but not within the CTV. T1PC failure was defined as a recurrence within the rChoNAA > 2 volume but not in the T1PC volume.

### Statistics

One-way ANOVA with multiple comparison tests was performed to evaluate volumetric differences between the varying imaging modalities. Actuarial analyses were performed via Kaplan–Meier methods. All statistics were performed in GraphPad Prism version 9.3.1 for PC (San Diego, CA).

## Results

### Patient baseline demographics

Median age of patients was 51.5 years old (Table 1). Most patients were IDH wildtype (79%) and MGMT non-hypermethylated (71%). Most patients had some level of residual disease after their primary surgery (64.3%) and patients had a median of 2 progressions prior to study. Just over 35% of patients had Eastern Cooperative Oncology Group (ECOG) performance scores of 2 or more. Median time from diagnosis was 13.8 months, and median time from last day of radiation treatment as part of trimodality therapy was 11.5 months.

**Table 1:** Baseline patient characteristics

Characteristic	n (%)
Age (median, years)	51.5
Gender	
Male	5 (36)
Female	9 (64)
IDH status	
Wildtype	11 (79)
Mutant	3 (21)
MGMT status	
Not hypermethylated	10 (71)
Hypermethylated	3 (21)
Indeterminant	1 (7)
Resection status	
Gross total resection	5 (36)
Subtotal resection	7 (50)
Biopsy only	2 (14)
ECOG performance status	
0	5 (36)
1	4 (29)
2	2 (14)
3	3 (21)
Number of recurrences (median)	2
Months from diagnosis (median)	13.8
Months from first RT start (median)	11.5

### Volumetric analyses

**Fig. 2:**
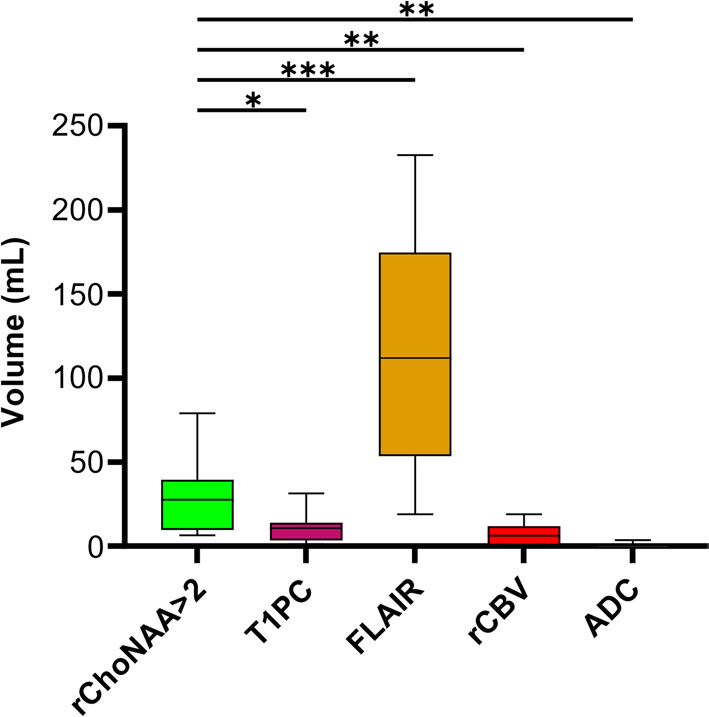
Volumes of contrasting imaging modalities. Comparison of the rChoNAA > 2, T1PC, T2/FLAIR, rCBV, and ADC volumes from 14 patients with recurrent GBM. Repeated measures one-way ANOVA with multiple comparisons shows significant differences between rChoNAA > 2 volume and other volumes. Box-and-whisker plots display the maximum, 75th percentile, mean, 25th percentile, and minimum: *P ≤ 0.05, **P ≤ 0.01, and ***P ≤ 0.001

rChoNAA > 2 volumes (mean 27.6 cc, range 6.6–79.1 cc) were significantly different from all other imaging modalities (Fig. 2). T1PC volumes had a mean of 10.7 cc (range 1.2–31.4 cc). The mean overlapping and non-overlapping volumes of rChoNAA > 2 and T1PC were 4.9 cc (range 1.1–15.7 cc) and 29.2 cc (range 4.7–79.1 cc), respectively. rChoNAA > 2 volumes were 287% larger (range 23% smaller to 873%) than T1PC on a per patient basis. Mean T2/FLAIR volumes were much larger than all other modalities at an average of 111.7 cc (range 19.0–233.7 cc). In comparison, rCBV (mean 6.2 cc, range 0.2–19.1 cc) and ADC volumes (mean 0.8 cc, range 0–3.7 cc) were miniscule.

**Fig. 3:**
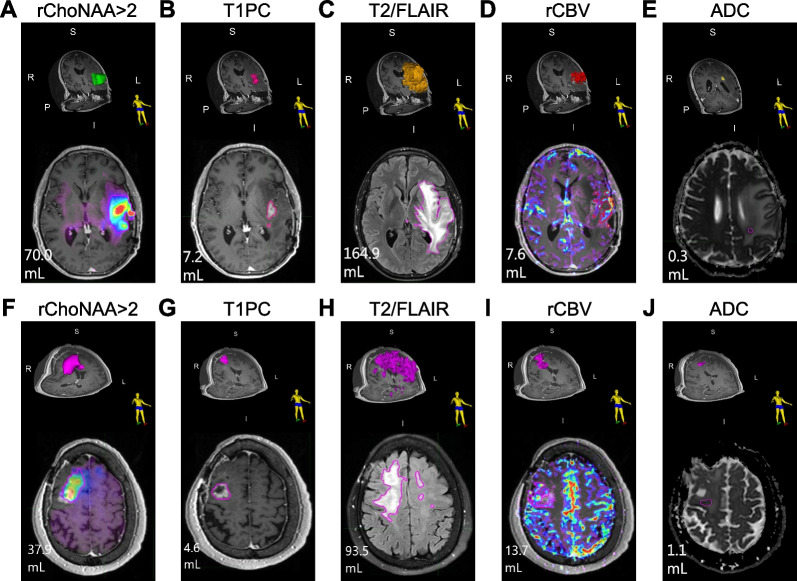
Example patients in selected axial slice and representative 3-dimensional volumetric contour. **A**–**E**. Example contours for patient with rGBM show rChoNAA > 2 volume, T1PC volume, T2/FLAIR volume, rCBV volume, and ADC volume. Pink contours indicate the areas of rChoNAA > 2, contrast enhancement, FLAIR hyperintensity, rCBV elevation, and diffusion restriction. **F–J** Example contours from a separate patient with rGBM shows rChoNAA > 2 volume, T1PC volume, T2/FLAIR volume, rCBV volume, and ADC volume. Pink contours indicate the areas of rChoNAA > 2, contrast enhancement, FLAIR hyperintensity, rCBV elevation, and diffusion restriction. I: inferior, L: left, P: posterior, R: right, S: superior, mL: milliliter

Example patient contours show rChoNAA > 2 volume was 70.0 cc (Fig. 3A), much larger than the T1PC volume of 7.2 cc (Fig. 3B). T2/FLAIR volumes were much larger than all other modalities at 164.9 cc (Fig. 3C). rCBV volumes (7.6 cc) were similar to T1PC, but the regions delineated were unhelpful for target volume identification (Fig. 3D). Similarly, ADC maps were unhelpful for target delineation (Fig. 3E). For a second example patient, the rChoNAA > 2 volume was 37.9 cc (Fig. 3F), much larger than the T1PC volume of 4.6 cc (Fig. 3G). T2/FLAIR volumes were the largest at 93.5 cc (Fig. 3H). Again, rCBV (Fig. 3I) and ADC maps (Fig. 3J) were very small compared to rChoNAA > 2.

### Patterns of failure

**Fig. 4:**
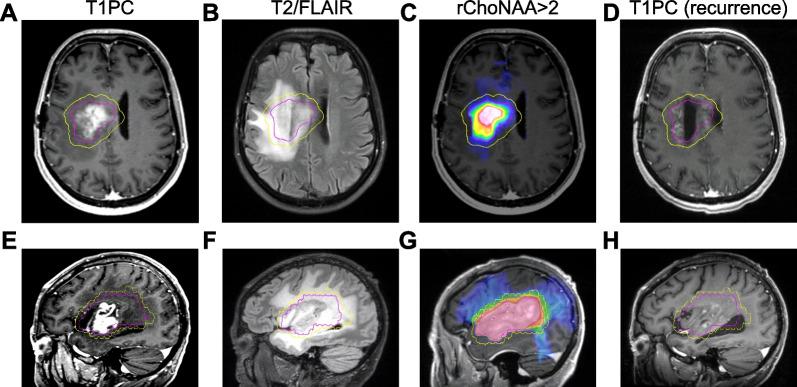
Two patients with recurrence within rChoNAA > 2 but outside of T1 post-contrast delineated GTV. **A** Axial pre-treatment T1PC from patient with rGBM treated with re-RT. Yellow outlines the CTV and magenta contour outlines the pre-treatment rChoNAA > 2 volume. **B** Pre-treatment T2/FLAIR. **C** rChoNAA > 2 overlaid pre-treatment T1PC. **D** Post-treatment T1PC. An area of recurrence is seen medially that was identified by the initial rChoNAA > 2 maps. **E** Sagittal pre-treatment T1PC from a separate patient with rGBM treated with re-RT. Yellow outlines the CTV and magenta contour outlines the pre-treatment rChoNAA > 2 volume. **F** Pre-treatment T2/FLAIR. **G** rChoNAA > 2 overlaid pre-treatment T1PC. **H** Post-treatment T1PC. Area of recurrence posteriorly was largely included in the rChoNAA > 2 volume but not T1PC volume. Note that areas of recurrence are not avidly enhancing due to concurrent bevacizumab administration

**Fig. 5:**
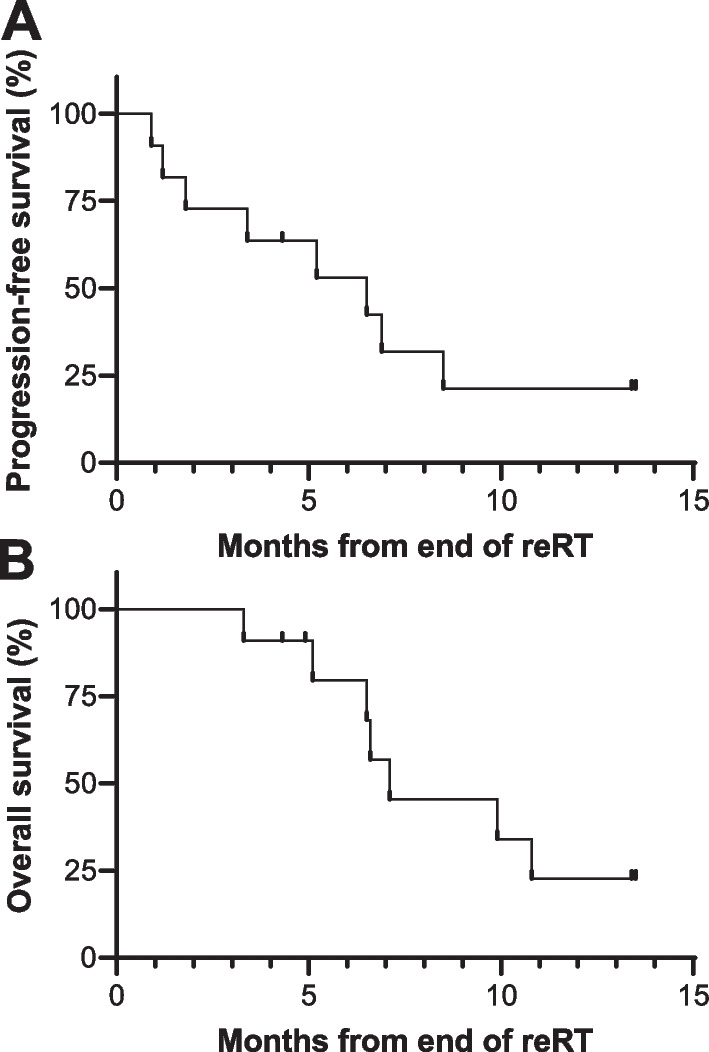
Progression-free and overall survival for patients receiving re-RT. **A** Kaplan–Meier graph showing progression-free survival for all patients who underwent re-RT. Median PFS = 6.5 months. **B** Kaplan–Meier graph showing overall survival for all patients who underwent re-RT. Median OS = 7.1 months

Of the 14 patients scanned, 11 received re-RT. Reasons for not proceeding with re-RT were rapid performance status decline and hospice (n = 2) or patient preference for systemic therapy (n = 1). For these 11 patients, median follow-up was 8.0 months (range 4.9–16.3 months). Out of the 11 patients, 8 failed in-field. Three out of the eight in-field failures were outside the T1PC delineated GTV, but within the rChoNAA > 2 delineated GTV (Fig. 4). Median survival time was 7.1 months and median progression-free survival was 6.5 months (Fig. 5). No grade 3 or higher toxicity were observed that were probably or definitely related to RT.

## Discussion

To our knowledge, this is the first study of sMRI based re-RT targeting in rGBM. We re-demonstrated that FLAIR hyperintense volumes are often large in rGBM. In upfront RT of GBM, the FLAIR hyperintense volumes are often treated due to high suspicion for disease [27]. However, in rGBM FLAIR is typically not used to guide re-RT on most studies because of fears of toxicity, and because much of the FLAIR volume is suspected to be benign from prior RT or other causes such as vasogenic edema. Conversely, in this series the T1PC volumes typically used to guide re-RT are about 90% smaller than FLAIR hyperintensity. Complementary to the standard imaging, we found that sMRI-derived rChoNAA > 2 volumes typically confirmed active disease within the T1PC volumes and then extended significantly into the FLAIR volume, identifying larger areas of non-enhancing malignancy within the FLAIR hyperintensity volume. The rChoNAA > 2 delineated GTVs identified areas of malignancy that would have been missed by RT based on T1PC-based target delineation. After treatment, all patients who failed RT recurred within the CTV delineated by the summed volumes of both T1PC and rChoNAA > 2. All this suggests that the addition of rChoNAA > 2 adds complementary value for demarcating high-risk rGBM targets and the current 35 Gy in 10 fraction dose in rGBM may be insufficient for durable local control.

A concern for re-RT of rGBM is radiation necrosis, particularly in the setting of dose escalation [28]. The risk of radiation necrosis can reach more than 24% when the cumulative dose is escalated to between 124 and 150 equivalent dose in 2 Gy fractions (EQD2) [29]. The treatment for radiation necrosis is bevacizumab, which also has activity in rGBM [30, 31]. This suggests that bevacizumab may prevent radiation necrosis leading to the limited toxicity observed in this study despite use of enlarged radiation fields compared to other studies. Indeed, a recent meta-analysis of 1399 patients who received reirradiation alone or in combination with bevacizumab found a significant decrease in the rate of radiation necrosis with the addition of bevacizumab [32]. As such, we have opened a prospective therapeutic trial (NCT05284643) of sMRI and bevacizumab based RT field size and dose escalation for rGBM.

No standard of care has been established in the management of rGBM, as no multi-center randomized trials have shown an overall survival benefit to any modality. Options for second line therapies include surgery, radiation, and systemic therapies. A systematic review and meta-analysis of 50 publications suggested a 6- month PFS benefit with re-RT; however, most studies included were level III evidence [33]. RTOG 1205 demonstrated an improved 6 month PFS (54% vs 29%, *P* = 0.001) and median PFS (7.1 vs 3.8 months, *P* = 0.051) with low (5%) frequency of severe acute toxicities when hypofractionated radiation is added to bevacizumab monotherapy [21]. Our goal is to build upon this PFS benefit towards an OS benefit by enhancing re-RT dose and targeting. While difficult to compare directly, the reported median PFS of 6.5 months in this study is from a separate patient population with rGBM treated with re-RT [21]. Our goal is to build upon this PFS benefit towards an OS benefit by enhancing re-RT dose and targeting. The outcomes in this study are favorable considering the higher proportion of pre-treated and poor performance status patients in our cohort. Systemic therapies alone demonstrated lower PFS, for example, with a median PFS of 3.5 months with bevacizumab in Checkmate 143, median PFS of 4.2 months with bevacizumab and lomustine in EORTC 26,101, and a median PFS of 3.4 months with bevacizumab and the viral agent VB-111 in GLOBE [34–36].

We also analyzed the potential value of rCBV and ADC in rGBM re-RT planning as they are often used to assist clinicians to identify rGBM and differentiate it from radiation necrosis [37]. Attempts have also been made to use these volumes to guide rGBM dose escalation in the upfront GBM setting [38]. Our study found no utility for re-RT targeting by rCBV/ADC given tiny identified volumes, though differences in technique might improve that capability.

The findings of this study must be viewed within the context of our limitations such as the study’s non-therapeutic nature, single institution non-randomized design, and limited number of patients. One concern is that while rChoNAA > 2 has been validated as a tumor marker in glioblastoma, that metabolite ratio may not be the best sMRI metric for delineation of rGBM. Still, we are encouraged by a radiology-pathology correlation study in rGBM demonstrating mean Cho/NAA ratios of 3.48 for tumor, 1.31 for radiation injury, and 0.79 for normal appearing white matter, declaring a ratio of 1.8 as a differentiator for tumor in 27 out of 28 patients tested [39].

## Conclusions

In summary, target volumes delineated by choline to *N*-acetyl-aspartate ratio greater than 2 generated from spectroscopic magnetic resonance imaging revealed areas of glioblastoma recurrence not identified using standard T1 post-contrast enhancing sequences alone. Treatment failures may be prevented by expanding treatment fields to cover these high-risk areas. Dose escalation may be necessary to impact failure rates within the treatment field. Future plans include radiologic-pathologic validation of sMRI for rGBM and a prospective trial to investigate the safety of enlarged and dose-escalated RT fields based on sMRI for rGBM.

## Data Availability

Data sharing is not applicable to this article as no datasets were generated or analysed during the current study.
